# Slow velocity of the center of pressure and high heel pressures may increase the risk of Sever’s disease: a case-control study

**DOI:** 10.1186/s12887-018-1318-1

**Published:** 2018-11-19

**Authors:** David Rodríguez-Sanz, Ricardo Becerro-de-Bengoa-Vallejo, Daniel López-López, Cesar Calvo-Lobo, Eva María Martínez-Jiménez, Eduardo Perez-Boal, Marta Elena Losa-Iglesias, Patricia Palomo-López

**Affiliations:** 1 Department, Faculty of Health, Exercise and Sport, European University of Madrid, Madrid, Spain; 2 Facultad de Enfermería, Fisioterapia y Podología UniversidadComplutense de Madrid, Madrid, Spain; 3 Research, Health and Podiatry Unit, Department of Health Sciences, Faculty of Nursing and Podiatry, Universidade da Coruña, Ferrol, Spain; 4 Nursing and Physical Therapy Department, Faculty of Health Sciences, Universidad de León, Ponferrada, León Spain; 5 Faculty of Health Sciences, Universidad Rey Juan Carlos, Alcorcon, Spain; 6 University Center of Plasencia, Universidad de Extremadura, Plasencia, Spain

**Keywords:** Velocity of the Centre of pressure, Apophysitis, Adolescent, Pressure, Equinus

## Abstract

**Background:**

This study determined if the body mass index, dynamic plantar-pressures, plantar surface contact-area, velocity of the centre of pressure (COP), gastrocnemius equinus, and gastrocnemius soleus equines are related to calcaneal apophysitis (Sever’s disease) in athletic children.

**Methods:**

This case-control study examined 106 boys enrolled in a soccer academy, including 53 with Sever’s disease and 53 age-matched healthy controls. The dynamic average and maximum peak plantar-pressures, plantar surface contact-area, and velocity of the COP were evaluated with a digital pressure sensor platform. Goniometry was used to measure the ankle dorsiflexion range of motion and thereby identify gastrocnemius equinus and gastrocnemius soleus equinus.

**Results:**

Participants with Sever’s condition had significantly higher BMI and peak plantar-pressures (maximum and average) at the heel (Cohen’s d > 3 for pressures) than the controls. Those with Sever’s disease also had significantly slower velocity of the COP (Cohen’s d > 3). Boys with Sever’s disease were also 8 times more likely to have bilateral gastrocnemius equinus than disease controls.

**Conclusions:**

High heel plantar pressure and low velocity of COP are related to Sever’s condition in boys, although it is not clear whether these factors predispose individuals to the disease or are consequences of the disease. Gastrocnemius ankle equinus could be a predisposing factor for Sever’s condition.

## Background

Calcaneal apophysitis is related to microtrauma at the bone-cartilage junction [1] resulting from overuse and repetitive movements during periods of rapid growth. Haglund first described calcaneal apophysitis. In 1912, Sever [2] described the condition, which was later given the name “Sever’s disease.”

Calcaneal apophysitis is a cartilaginous growth centred on the point where the Achilles tendon inserts into the heel [3–5]. The heel pain involved in Sever’s disease limits sports activities and may interfere with other different activities of daily living. The disease is self limiting because the calcaneal cartilage eventually decreases at the age of about 14 years in girls and 16 years in boys, when the calcaneus ossifies completely [6].

Calcaneal apophysitis or Sever’s disease has been defined as the most prevalent cause of heel pain in athletic children related to the activation of the triceps surae muscle [7, 8]. The etiological factors for Sever’s condition are unclear and mostly speculative [9]. Researchers have hypothesized both intrinsic and extrinsic potential factors. Possible intrinsic factors include a limited range of ankle dorsiflexion motion [10, 11] and high static [12] and dynamic plantar pressures [13] at the heel. Potential extrinsic factors include activities with high foot impact, footwear, and sports activities on hard surfaces [8, 14, 15]. However, the empirical evidence for most of the hypothesized contributing factors is limited or absent [16].

To address this empirical gap, we conducted a matched case-control study on athletic children to examine factors that are evaluated by clinicians in the management of heel pain and their associations with Sever’s disease. Specifically, we investigated the body mass index (BMI), dynamic plantar-pressures, plantar surface contact-area, velocity of the centre of pressure (COP) during walking, gastrocnemius equinus, gastrocnemius soleus equinus, and their relationships to Sever’s disease. The research question for this study was:

How are BMI, dynamic plantar-pressures, plantar surface contact-area, velocity of the COP, gastrocnemius equinus, and gastrocnemius soleus equinus related to calcaneal apophysitis (Sever’s disease) in children?

## Methods

Subjects in this study were young athletes enrolled in a soccer academy who presented for a health screening at the end of the season. A total of 106 boys between the ages of 9 and 14 years were assessed for study eligibility. Participants were excluded if they had a neurological condition affecting either of the lower extremities or a recent history of foot and ankle trauma (including tendinitis, sprain, or any pathology other than Sever’s disease). All participants received the same amount of training hours and physical activity per week.

A total of 106 participants were enrolled in the study, including 53 boys with unilateral symptomatic Sever’s disease and 53 healthy children who never had heel pain and served as control participants. Clinical assessment for the diagnosis of Sever’s condition was carried out by the same podiatry doctor (RBBV). The main diagnostic criterion for calcaneal apophysitis was pain related to compression of the mediolateral calcaneus in the growth plate region [3]. The pain had to have a duration of at least 2 months, it had to be related to physical activity, and it had to be severe enough to stop normal physical activity [7].

The controls were selected from the pool of participants with random-number software (Epidat 3.1, Pan American Health Organization, Washington D.C., USA). The controls were individually matched to the cases by age and the assessed foot. No differences were found between or within the groups in terms of the time for sports participation and competition time, including after school sports activities and weekend training.

This case-control study conformed to the guidelines set forth in the Declaration of Helsinki and was approved by the research committee of Hospital La Princesa (Spain; code number 2828A). The parents of participating children gave informed written consent, and the participating children gave verbal assent prior to data collection. The investigator (DRS) who collected the data and performed testing for all participants had extensive experience in conducting physical examinations of the foot and ankle (over 10 years). The same testing equipment and procedures were used to assess both groups.

We measured each participant’s height using a tallimeter (QUIRUMED, Valencia, Spain) with the participant barefoot and the arms hanging at rest. We also weighed each participant with a scale (QUIRUMED 627-MPS200K, Valencia, Spain). The Silfverskiold test [17, 18] was performed by measuring the ankle-dorsiflexion range of motion using a clinical goniometer while the participant’s knee was alternately flexed and then extended.

For the flexed position, the participant laid on his back with the knee flexed at 90°. The amount of ankle dorsiflexion was defined as the angle between the plantar aspect of the heel (medially or laterally) and the tibia. Care was taken to maintain the subtalar joint in a neutral position to ensure that the ankle dorsiflexion was measured and not midfoot dorsiflexion (rocker bottom) or midfoot equinus (pseudoequinus) [18, 19].

Dynamic plantar pressure was measured with a pressure-sensor platform (Win-pod, Medicapteurs, Balma, Fra) embedded in a flat 5-m walkway. Each participant walked barefoot in the walkway at a normal self-selected speed beginning 2 m in front of the platform and ending 1 m behind it. The participants were told to look forward and not to focus on any particular region of the walkway so as not to target the platform. The starting position of each participant’s walk was adjusted after several practice trials so that one foot would land on the pressure platform during a normal stride. The long path allowed measurements to be recorded during steady-state gait, which ensured that the effect of acceleration and deceleration at the start and end of each walk was minimized.

After familiarization with the testing procedures, a trial was considered as valid when the following criteria were met: (1) the presence of a heel and toe strike pattern, (2) prescribed normal speed looking forward, and (3) no visible adjustment in gait pattern when crossing the plate [20–22]. Trials that did not have these characteristics were excluded from the analysis. Observations were made for the second step of a designated foot in a valid walking trial (the two-step method), and six measurements on each foot were collected from every participant [23, 24]. The subject did not know when a trial was acceptable and being registered.

Pressure-sensor measurements from the platform were accurate to the nearest 0.001 kg/ cm^2^. Although the platform has an auto-calibration system that runs each time it is turned on, a calibration was also performed by the manufacturer prior to the initiation of the study. We obtained a certificate of European approval (CE) for the platform to be used as a medical device. We calculated the BMI as the weight in kilograms divided by the square of the height in meters based on the Quetelet index equation.

The Winpod software produced pressure maps, which included measurements of the average peak pressure (g/cm^2^), maximum peak pressure (g/cm^2^), velocity (mm/s) of the COP, and plantar surface contact area (cm^2^) for each trial. The dynamic plantar-pressure outline for each participant was divided into four different sections: the heel, midfoot, forefoot, and toes. The heel, midfoot, and forefoot were marked as equal thirds of the plantar-pressures outline without the toes [23, 25].

When recorded with a force platform, the COP is defined as the centroid of the vertical force distribution on the ground plane [26]. In young healthy feet, the velocity of the COP has three characteristic peaks during the stance phase of the gait [27, 28]. The first peak occurs in the rearfoot region between 0 and 20% of the stance phase corresponding to the “loading” response of the gait [27]. The second peak occurs at approximately 35% of the stance phase, when the COP moves from the midfoot to the forefoot. The final peak is at 92% of the stance phase, when the heel lifts off.

In our analyses, we focused on the average peak pressure, maximum peak pressure, plantar surface contact area, and velocity of the COP at three points in the stance phase (20%, 35%, and 92%). The 20% and 35% measurements involved the heel, while the 92% measurements involved the forefoot. All measurements were recorded as the means of a participant’s six valid trials for a given foot.

Gastrocnemius equinus is present when the gastrocnemius muscle limits the ankle-dorsiflexion range of motion, and gastrocnemius soleus equines is present when the soleus muscle limits the ankle-dorsiflexion range of motion [19, 29]. Following prior investigators [24, 30], we defined gastrocnemius equinus as ankle dorsiflexion less than 10° in both knee-flexed and knee-extended positions. We defined gastrocnemius soleus equinus as the inability of the ankle to dorsiflex beyond a neutral position (remaining < 0°) when the knee is flexed or extended.

We computed the means, standard deviations, and 95% confidence intervals for age, height, weight, and BMI for the participants overall and for the cases and controls separately. We also noted which heels were the focus of the analysis (Table 1). We calculated the intraclass correlation coefficients to assess the between-trial reliability for each plantar platform variable. Landis and Koch^33^ gave the following interpretation of coefficient values: 0.20–0.40 = fair, 0.40–0.60 = moderate, 0.60–0.80 = substantial, and 0.80–10.00 = almost perfect reliability.

**Table 1:** Characteristics of boys with and without Sever’s disease

Characteristic	Total participants (*N* = 106)	Sever’s disease group (*n* = 53)	Control group (*n* = 53)	*P* value
Age in years^a^	10.73 ± 1.36 (10.47–10.99)	10.66 ± 1.50 (10.25–11.06)	10.81 ± 1.20 (10.48–111.14)	0.570
Height in cm^a^	142.99 ± 9.21 (141.23–144.74)	144.05 ± 10.75 (141.16–146.95)	141.92 ± 7.31 (139.95–143.89)	0.235
Weight in kg^a^	38.35 ± 7.50 (36.92–39.78)	40.21 ± 8.38 (37.95–42.47)	36.49 ± 6.03 (34.86–38.11)	0.005
BMI^a^	18.63 ± 2.24 (18.20–19.05)	19.18 ± 1.88 (18.68–19.69)	18.07 ± 2.44 (17.41–18.73)	0.009
Heel affected, right/left	80 right/26 left	40 right/13 left	40 right/13 left	–

^a^The cells in these rows show means±standard deviations, with 95% confidence intervals in parentheses

*Abbreviation*: *BMI* body mass ind

We used cross-tabulations and chi-squared tests to compare the prevalence of each type of equinus in the cases and controls. The Kolmogorov-Smirnov statistical test was carried out to assess the normality of the plantar platform measures. We used Cohen’s d (standardized mean difference) and a student’s t-test to compare each of the plantar platform measures between the cases and controls. Cohen^34^ recommended the following interpretations of d: < 0.1 = trivial difference, 0.1–0.3 = small difference, 0.3–0.5 = moderate difference, and > 0.5 = large difference.

One-factor ACOVA (factor: Sever’s disease; covariables: height and weight) was carried to examine the influence of weight and height on the velocity of COP in both groups. For all analyses, we set the threshold for statistical significance as *p* < 0.01. All the statistical analyses were done using SPSS 19.0 (Chicago, IL, USA).

## Results

The Kolmogorov-Smirnov test showed a normal distribution in all pressure platform variables. All coefficients were greater than 0.89, indicating very high between-trial reliability. Table 2 shows the results of the pressure platform variables measured with the heel at 20% and 35% of the stance phase, respectively. The average and maximum peak pressures participants were many times higher in those with Sever’s disease than the controls (*p* < 0.001). Despite the very large differences, the cases and controls had very similar plantar surface contact areas on average. Those with Sever’s disease also had much lower velocity of the COP than the controls (*p* < .001).

**Table 2:** Pressure platform measurements at the heel at 20%, 35% and 92% of the stance phase for boys with Sever’s disease and controls

Variable	Total (*N* = 106) Mean ± SD (95% CI)	Sever’s disease (*n* = 53) Mean ± SD (95% CI)	Control group (*n* = 53) Mean ± SD (95% CI)	*P* value	Cohen’s d
Average peak pressure at the heel at 20% of the stance phase (g/cm^2^)	3985.15 ± 3493.28 (3320.14–4650.16)	7273.26 ± 1610.49 (6839.68–7706.84)	697.05 ± 89.03 (673.08–721.02)	< 0.001	4.083
Maximum peak pressure at the heel at 20% of the stance phase (g/cm^2^)	4620 ± 3902.92 (3877.40–5363.39)	8451.09 ± 914.81 (8204.81–8607.38)	789.70 ± 93.80 (764.44–814.95)	< 0.001	8.374
Velocity of the COP at the heel at 20% of the stance phase (mm/s)	42.17 ± 15.42 (39.24–45.11)	27.74 ± 3.94 (26.68–28.80)	56.61 ± 6.31 (54.91–58.31)	< 0.001	4.575
Plantar surface contact area at the heel at 20% of the stance phase (cm^2^)	30.08 ± 3.59 (29.40–30.76)	30.11 ± 3.52 (29.16–31.06)	30.05 ± 3.70 (29.05–31.05)	0.935	0.017
Average peak pressure at the heel at 35% of the stance phase (g/cm^2^)	3550.67 ± 3141.50 (2952.63–4148.72)	6404.82 ± 1818.88 (5915.14–6894.50)	696.52 ± 117.92 (664.78–728.27)	0.001	3.138
Maximum peak pressure at the heel at 35% of the stance phase (g/cm^2^)	4448.57 ± 3716.58 (3741.05–5156.10)	7990.23 ± 1520.87 (7580.78–8399.68)	906.92 ± 97.58 (880.65–933.19)	0.001	4.657
Velocity at the heel at 35% of the stance phase (mm/s)	50.34 ± 16.92 (47.12–53.56)	35.51 ± 5.87 (33.93–37.09)	65.17 ± 9.77 (62.54–67.80)	0.001	3.035
Surface at the heel at 35% of the stance phase (cm^2^)	52.27 ± 6.02 (51.12–53.41)	51.30 ± 4.33 (50.13–52.46)	53.24 ± 7.24 (51.29–55.19)	0.090	0.267
Average peak pressure at the forefoot at 92% of the stance phase (g/cm^2^)	982.97 ± 312.21 (923.53–1042.41)	1200.14 ± 303.29 (1118.49–1281.80)	765.80 ± 93.28 (740.68–790.91)	0.001	1.432
Maximum peak pressure at the forefoot at 92% of the stance phase (g/cm^2^)	1248.85 ± 436.62 (1165.73–1331.97)	1560.41 ± 422.90 (1446.56–1674.27)	937.29 ± 90.71 (912.87–961.71)	0.001	1.473
Velocity of the COP at the forefoot at 92% of the stance phase (mm/s)	33.31 ± 5.44 (32.27–34.35)	34.59 ± 5.31 (33.16–36.02)	32.03 ± 5.32 (30.59–33.46)	0.014	0.481
Surface at the forefoot at 92% of the stance phase (cm^2^)	43.19 ± 4.81 (42.28–44.11)	42.96 ± 1.11 (41.85–44.07)	43.43 ± 5.44 (41.96–44.89)	0.616	0.086

Figure 1.1 and 1.2 show the pattern of the results for 20% and 35% of the stance phase, respectively, for a representative case and a representative control. The load in the heel area was significantly higher for the subject with Sever’s disease than the control. The line of the COP for the subject with Sever’s disease was also less straight and longer than that for the control, indicating more time in contact with the floor and higher load.

**Fig. 1:**
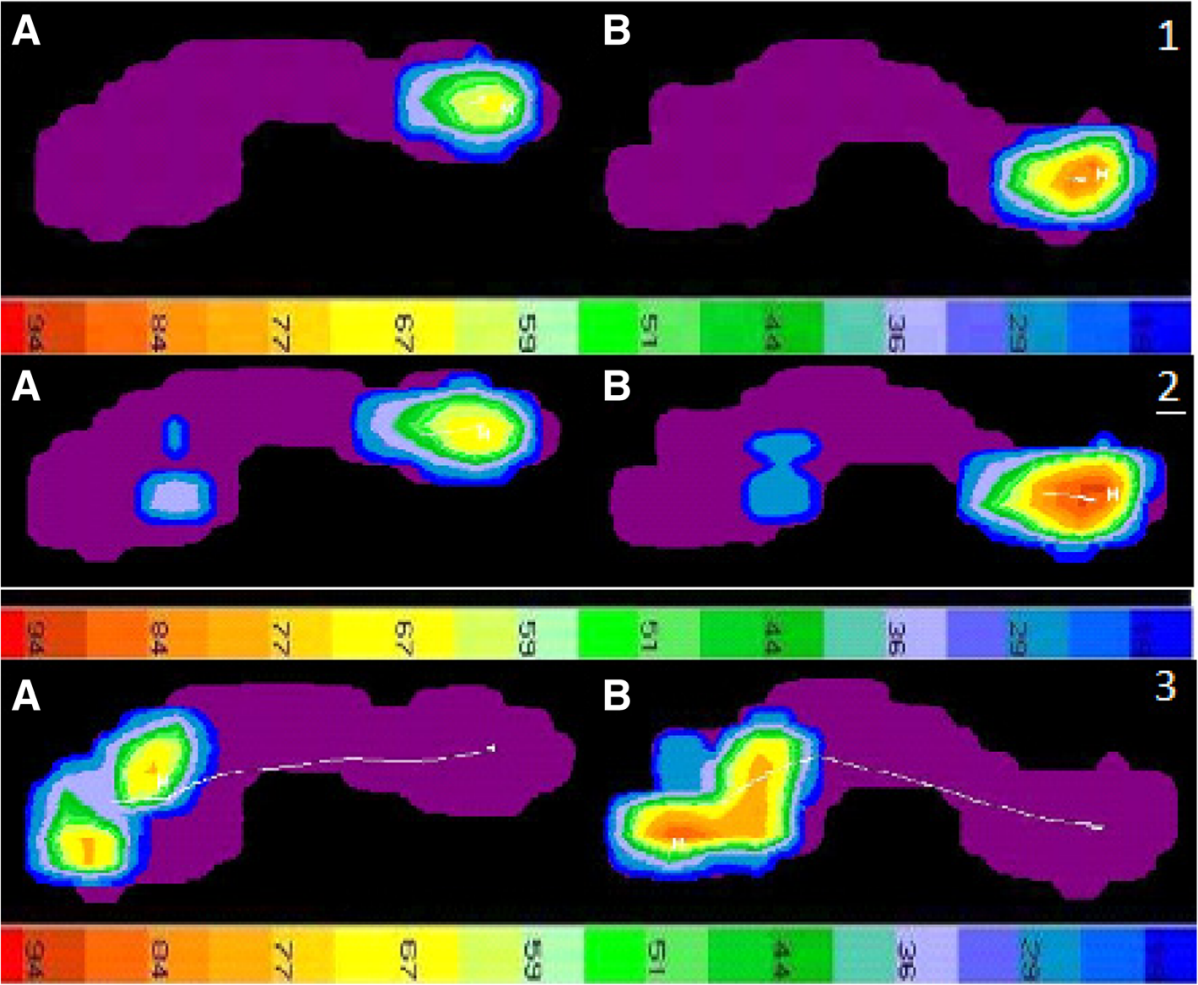
**1**. Distribution of pressure at 20% of the stance phase of gait for a representative control boy (A) and a representative boy with Sever’s disease (B). The scale at the bottom indicates pressure (g/cm^2^). The white lines show the COP for each group. **2**. Distribution of pressure at 35% of the stance phase of gait for a representative control boy (A) and a representative boy with Sever’s disease (B). The scale at the bottom indicates pressure (g/cm^2^). The white lines show the COP for each group. Figure **3**. Distribution of pressure at 92% of the stance phase of gait for a representative control boy (A) and a representative boy with Sever’s disease (B). The scale at the bottom indicates pressure (g/cm^2^). The white lines show the COP for each group

Table 2 shows the results for the pressure platform variables measured at the forefoot at 92% of the stance phase, when the heel has lifted off. Those with Sever’s disease had higher average and maximum peak pressures than the controls (*p* < 0.001). Nonetheless, the cases and controls had very similar plantar surface contact areas on average. Those with Sever’s disease also had moderately lower velocity of the COP than the controls, although the difference was not significant (*p* = 0.014). Figure 1.3 shows the results for 92% of the stance phase for a representative case and a representative control.

The one-factor ANCOVA results obtained for the velocity at 20% of the stance phase shows no influence of height and weight on the velocity of COP. We found significant differences (*p* < 0.01) between the velocity of the Sever group (27.74 ± 3.94 mm/seg) and non-Sever group (55.64 ± 8.22 mm/seg). For 35% of the stance phase, we found no influence of weight and height and obtained significant differences (*p* < 0.01) between the velocity of the Sever group (34.99 ± 1 mm/seg) and non-Sever group (66.21 ± 0.68 mm/seg). Similarly, the results for 92% of the stance phase show no influence of weight and height and significant differences (*p* < 0.01) for velocity between the Sever group (34.59 ± 5.3) and non-Sever group (31.48 ± 4.66). The same trend was found for velocity values at 100% of the gait process. We found no influence of height and weight and significant differences (*p* < 0.01) between the velocity of the Sever group (69.3 ± 0.65) and non-Sever Group (66.46 ± 0.44).

Those with Sever’s disease had a higher BMI and weighed more on average than the controls. The cases also had lower ankle range of motion than the controls, as indicated by a higher prevalence of gastrocnemius equinus. Furthermore, those with Sever’s disease had much higher plantar pressures and lower velocity of the COP at 20%, 35%, and 92% of the stance phase than the controls on average, although the plantar surface contact areas were similar between groups.

## Discussion

Our results are consistent with prior research, which also indicated that static and dynamic plantar pressures [12, 13] were higher in children with Sever’s disease than in healthy children. To our knowledge, no prior study has investigated the velocity of the COP during walking in relation to Sever’s disease. In our study, the heels of those with Sever’s disease supported higher average and peak pressures for more time than the heels of the controls. The greater amount and duration of pressure on the heel could lead to secondary injury of the vulnerable immature calcaneal cartilage. Jahss and colleagues showed that if the force or repetition of force is not attenuated to below a critical level, tissue damage can result, with healing responses leading to further structural changes and alterations to the tissue mechanics. These changes can modify forces on the heel during the gait and lead to repetitive microtrauma [3, 8].

Although gastrocnemius equinus and gastrocnemius soleus equinus are common in asymptomatic subjects, equinus may be a causative factor in many other foot and ankle pathologies, including plantar fasciitis, pes planus, hallux abducto valgus, Achilles tendinosis, Charcot’s midfoot collapse, and diabetic ulcerations [24]. The mechanism by which ankle equinus may influence calcaneal apophysitis or Sever’s disease is unclear [18]. Gastrocnemius equinus might lead to mechanical overloading in the symptomatic heel as a direct consequence of soft tissue tightness. Tight triceps surae can cause excessive tension through the Achilles tendon and increase the traction on the apophysis, which may lead to Sever’s disease due to an increased tractional effect [1, 8, 11].

One important limitation of this study is that with our case-control design, we cannot assess whether the high plantar pressures and low velocity of the COP are possible causes or consequences of Sever’s disease. Prospective and longitudinal trials are needed to shed light on the exact etiological factors of Sever’s disease. Additional work is also needed to investigate whether biomechanical factors beyond those assessed may be associated with diseases such as flat foot, pronated foot, and pes cavus, among other biomechanical conditions of the lower limbs. Other data-collection protocols for the plantar pressure variables might have produced different results. Nonetheless, our measurements displayed very high inter-trial reliability within participants. Furthermore, measurements of the ankle-dorsiflexion range of movement can be highly variable within and between raters. It would be beneficial to obtain a standardised approach for the measurement of ankle joint dorsiflexion. However, reliability is higher for gastrocnemius ankle motion than for gastrocnemius soleus ankle motion. In regard to the subtalar-joint neutral validity and reliability, the palpation technique as part of the subtalar joint axis location and rotational equilibrium theory proposed by Kirby is a reliable and valid clinical tool. Experience in performing the palpation technique has a positive influence on the accuracy of the results [31].

## Conclusion

High heel plantar pressure and low velocity of COP are associated with Sever’s condition in boys. However, it is not clear whether these factors predispose individuals to the disease or are consequences of the disease. Gastrocnemius ankle equinus may also be a predisposing factor for Sever’s condition.

### Practical implications

- Clinical screening was performed with a non-invasive diagnosis tool (force platform) to examine high heel plantar pressure of COP and its association with Sever’s disease.
- Screening was performed with a non-invasive diagnosis tool (force platform) to examine the association between low velocity of COP and Sever’s disease.
- Gastrocnemius ankle equinus may be a predisposing factor for calcaneal apophysitis or Sever’s condition in children.

## Abbreviations

BMI: Body mass index
CE: European approval
COP: Center of pressure
